# Comparing information derived on food habits of a terrestrial carnivore between animal-borne video systems and fecal analyses methods

**DOI:** 10.1093/jmammal/gyac101

**Published:** 2022-11-25

**Authors:** Shiori Tezuka, Mii Tanaka, Tomoko Naganuma, Kahoko Tochigi, Akino Inagaki, Hiroaki Myojo, Koji Yamazaki, Maximilian L Allen, Shinsuke Koike

**Affiliations:** Faculty of Agriculture, Tokyo University of Agriculture and Technology, Fuchu, Tokyo 183-8509, Japan; Faculty of Agriculture, Tokyo University of Agriculture and Technology, Fuchu, Tokyo 183-8509, Japan; Institute of Global Innovation Research, Tokyo University of Agriculture and Technology, Fuchu, Tokyo, 183-8509, Japan; United Graduate School of Agricultural Science, Tokyo University of Agriculture and Technology, Fuchu, Tokyo, 183-8509, Japan; United Graduate School of Agricultural Science, Tokyo University of Agriculture and Technology, Fuchu, Tokyo, 183-8509, Japan; United Graduate School of Agricultural Science, Tokyo University of Agriculture and Technology, Fuchu, Tokyo, 183-8509, Japan; Faculty of Regional Environmental Science, Tokyo University of Agriculture, Setagaya, Tokyo 156-8502, Japan; Illinois Natural History Survey, University of Illinois, Champaign, Illinois, 61820, USA; Institute of Global Innovation Research, Tokyo University of Agriculture and Technology, Fuchu, Tokyo, 183-8509, Japan

**Keywords:** fecal analysis, foraging behavior, individual difference, *Ursus thibetanus*, video analysis

## Abstract

In recent years, animal-borne video cameras have been used to identify the food habits of many species. However, the usefulness and difficulties of identifying food habits from animal-borne video cameras have not been sufficiently discussed in terrestrial mammals, especially large omnivores. The aim of this study is to compare the video analysis of foraging behavior by Asian black bears (*Ursus thibetanus*) acquired by camera collars with estimates from fecal analysis. We attached GPS collars equipped with video cameras to four adult Asian black bears in the Okutama mountains in central Japan from May to July 2018 and analyzed video clips for foraging behavior. Simultaneously, we collected bear feces in the same area to determine food habits. We found that using video analyses was advantageous to recognize foods, such as leaves or mammals, that were physically crushed or destroyed while bears chewed and digested foods, which are difficult to identify to species using fecal analyses. On the other hand, we found that camera collars are less likely to record food items that are infrequently or quickly ingested. Additionally, food items with a low frequency of occurrence and short foraging time per feeding were less likely to be detected when we increased the time between recorded clips. As one of the first applications of the video analysis method for bears, our study shows that video analysis can be an important method for revealing individual differences in diet. Although video analysis may have limitations for understanding the general foraging behavior of Asian black bears at the present stage, the accuracy of food habit data from camera collars can be improved by using it in combination with established techniques such as microscale behavior analyses.

The food habits of wildlife provide fundamental information required for understanding the ecology of individual species and functional components of ecosystems, such as how material and energy flow through food webs (e.g., Morrison et al. 1998). Historically, the food habits of a wide variety of terrestrial carnivore species have been studied using either direct observations of their foraging behavior or fecal analysis, but there are limitations to both methods. For example, it is difficult to directly observe food habits of many species, including those using dense forest habitat, species with nocturnal activity patterns, and otherwise cryptic or cautious species. Fecal analyses, while commonly used, are subject to differences in digestibility of ingested foods, leading to the overestimation or underestimation of certain kinds of food items (Litvaitis 2000).

In recent years, with advancements in technology, video cameras are now capable of being mounted on transmitters for various species of wild animals in order to record and observe their behavior (Kooyman 2007; Moll et al. 2007; Wilmers et al. 2015). Recordings made of feeding behavior have led to quantitative analyses and new knowledge of feeding behavior of marine mammals (Bowen et al. 2002; Pagano et al. 2018), reptiles (Heithaus et al. 2002; Arthur et al. 2007; Burkholder et al. 2011), and sea birds (Watanabe and Takahashi 2013). Heithaus et al. (2002) compared feeding behavior as assessed using video recordings vs. stomach contents. Further advancements in technology, including miniaturization of devices, have allowed for increasing research using video cameras mounted on terrestrial mammal species (e.g., Loyd et al. 2013; Hernandez et al. 2018).

In many cases, when a video camera built into a transmitter or mounted on a collar (hereafter, referred to as a camera collar) is mounted on terrestrial mammals, it is possible to capture images of food being eaten. Accordingly, some studies have attempted to carry out analyses of food habits based on video clips (hereafter, clips) of their feeding behavior. Many of these prior studies have focused on herbivorous ungulates (Beringer et al. 2004; Lavelle et al. 2012, 2015; Viejou et al. 2018). One advantage of using clips is that plant species that are difficult to identify by conventional fecal analyses may be identified—importantly, the accuracy of identification from clips has been demonstrated to be adequate when checked against DNA barcoding results (Newmaster et al. 2013), suggesting that camera collars are useful for carrying out analyses of food habits for ungulates. On the other hand, studies of large, terrestrial carnivorous mammals have been more limited—studies of terrestrial bears to date using data from collar cameras have focused on their predation on ungulates (Bowersock et al. 2015; Brockman et al. 2017), and in those studies, only the frequency of predation foraging behavior was estimated. The efficacy of using camera collars to study the food habits of large, terrestrial mammals other than ungulates warrants further investigation.

Asian black bears (*Ursus thibetanus*) are omnivorous but primarily eat plant materials. Most past studies of their food habits have been analyzed using fecal analyses. A limitation of this method is that the leaves of many herbaceous and woody plants, as well as meat, are destroyed beyond recognition during chewing and cannot be adequately identified (Koike 2010; Koike et al. 2013). A recent study (Naganuma et al. 2020, 2022) revealed that individual bears in a population may select different types of food items depending on traits such as age, sex, and morphology, but this study was based on stable isotope ratio analysis, so specific food item identification was not possible. DNA analysis of fecal contents may reveal individual differences in specific food items, although this has not yet been done for Asian black bears. Furthermore, differences in habitat selection and behavioral attributes are known to exist among individuals (e.g., Takahata et al. 2017). Perhaps there is a close relationship between individual differences in diet and individual differences in behavior, but the causal aspect of this relationship is unknown.

Camera collars may provide greater insight into the relationship between each of these attributes and selection of food items. By using camera collars (hereafter, video analysis), and acquiring both macroscale (GPS) and microscale behavioral data, it may be possible to identify food items that are destroyed beyond recognition during chewing, and to assess behavioral patterns that affect individual differences in foraging behavior.

In this study, we investigated the following objectives and hypotheses with the aim of assessing the characteristics, effectiveness, and limitations of video analysis from camera collars to understand foraging behavior in terrestrial large carnivores. Our first objective was to compare the fecal analysis method with the video analysis method, and to examine characteristics of food items captured by each method. We hypothesized that video foraging analysis would better identify more easily digestible herbaceous and woody plants than the fecal analysis method. Next, with a camera collar, shorter times between clips will more quickly exhaust battery and memory capacity and thus limit the total monitoring period of a collar, decreasing the amount of data on foraging behavior that can be acquired. Therefore, our second objective was to examine the effectiveness of video analysis in assessing foraging habits and food item selection as time between clips is increased. We hypothesized that two attributes—food items with a low frequency of occurrence, and less time that an individual spends continually foraging for an item at a time—would be less likely to be detected when the times between clips were increased.

## Materials and Methods

### Study area

**Fig. 1.**
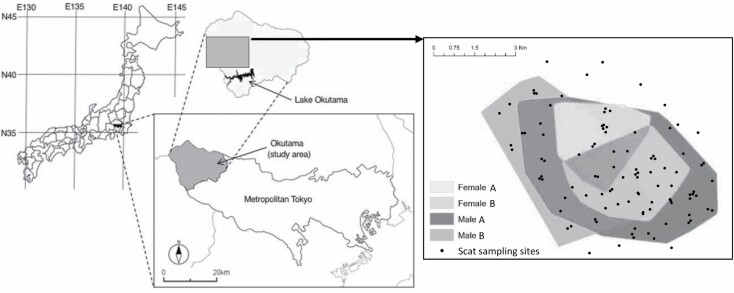
—Map of the study area, the home ranges (the 100% minimum convex polygon) of each bear, and scat sampling locations in Okutama, Tokyo, Japan.

We conducted our study in Okutama Town (35°48ʹN, 139°5ʹE), the westernmost suburb of Tokyo (Fig. 1). The climate of the study area is linked to the Pacific Ocean, with heavy rainfall in summer and little snow in winter. The mean annual precipitation is 1,510 mm and the mean annual temperature is 12.4°C, ranging from 0.6°C in January to 24.2°C in August (data from 2006 to 2017; Japan Meteorological Agency 2021).

The study area is mostly covered with a mixed mosaic of forest vegetation (Koike et al. 2008b; Okutama Town 2014). Conifer plantations covering 50% of the area include the tree species Japanese cedar (*Cryptomeria japonica*), Japanese cypress (*Chamaecyparis obtuse*), and Japanese larch (*Larix kaempferi*), natural forest cover 40% of the area, and agriculture or urban areas cover 10%. In the conifer plantations, Japanese cedar covers 65% of the area and Japanese cypress covers 25% of the area. Japanese larch occurs mainly above 1,000 m elevation and covers 10% of the area. The natural forest is dominated by broadleaf trees (*Castanea crenata* and *Quercus serrata*) in the lower mountain zone (400–500 m above sea level [a.s.l.]), by broadleaf trees (*Q. crispula*, *C. crenata*, and *Fagus crenata*) in the middle zone (500–1,500 m a.s.l.), and by conifer trees (*Abies homolepis* and *Tsuga diversifolia*) in the upper zone (1,500–1,800 m a.s.l.).

### Bear capture

We captured Asian black bears in the study area from May to June 2018 using five barrel traps baited with honey set within 2.5 km of each other. We immobilized the trapped bears with tiletamine hydrochloride and zolazepam hydrochloride (Virbac, Carros, France; 8 mg/kg of estimated body weight). We documented basic body measurements and extracted a premolar for age determination. We then fit each bear with microchips and GPS collars mounted with digital cameras (GPS Vertex Lite Collars; Vectronic Aerospace GmbH, Berlin, Germany, 29.97 fps, pixel size: 1,920 × 9.97, dimensions: 70 × 70 × 84 mm, 1,200 g). Due to the weight of the GPS collars, we only included bears weighing >40 kg in this study. We dropped the collars using a remote control at the end of the programmed lives of the cameras. We performed all our bear capture and handling in accordance with the guidelines for animal research established by the Mammal Society of Japan (2009).

### Camera programming

We selected the “sampling interval” (the period that cameras were recording based on the time of day when bears are active daily during this season; Yamazaki et al. 2008), while accounting for amount of memory and camera battery life (18 h). We programmed each camera collar to record a 10-s video clip and the GPS location of the bear every 15 min, with a duty cycle of 13 h on (05:00–17:45; total 52 clips per day) and 11 h off based on the sunup and sundown times during June and July. The data were stored onboard the devices, and then recovered collars were returned to the manufacturer for downloading of the digital video clips. The video clips were processed by sorting them into a single video montage.

### Video data classification and video analysis

Due to concern that the behavior of the bears may have been influenced by initial attempts to remove the collar immediately after capture, for the analysis we used the data from 05:00 the day after capturing bears until 17:45 the day before remotely dropping their collars.

We created a primary classification for each clip that was based on seven behavioral classifications, including; “traveling by land,” “sleeping,” “resting,” “foraging,” “sniffing something (excluding food items),” “other,” and “unclear.” “Foraging” included feeding behavior, as well as searching for food behaviors such as digging in the ground, breaking decayed trees, stripping tree bark, and breaking branches on trees. As bears can forage on berries or ants and maintain a normal walking pace, if bears were observed to be foraging on something while traveling we classified the behavior as foraging. “Other” included behaviors such as drinking, tree rubbing, and climbing a tree. “Unclear” included the clips that could not be determined due to issues such as hair covering the camera.

As our objectives for this study were primarily to research food habits, we identified the food items from each clip as best as possible (fruits, wood plants, and animal materials were identified by S. Koike; grass plants were identified by M. Yoshikawa) and used three steps to calculate their relative importance. First, we calculated the frequency of each food item appearing in the clips for each individual bear (hereafter, shooting frequency %). Second, in order to understand the effect of different clip shooting interval times on whether or not food items were documented, we calculated the shooting frequency of each food item shot in each clip using 30-min intervals (using clips of 00 min and 30 min every hour), 60-min intervals (using clips of 00 min every hour), and 120-min intervals (using clips of 00 min at even times). Third, we also recorded the number of continuous clips (clips collected in 15-min intervals and pooled from all individuals) when the same food item was consumed at the same location (hereafter, continuous clips).

### Fecal analysis

We collected scat samples (*n* = 102) at the same times and in the same places as our bear tracking from late May to early July in 2018. We collected scats via targeted searches around clusters of locations where GPS-collared bears had spent at least 4 h (*n* = 61, 59.8%) opportunistically while handling captured bears and on the way to the GPS clusters, and at several backcountry sites as part of a concurrent study on other topics (Fig. 1). Additionally, the results of the video analysis showed that all collared individuals spent an average of more than 40% of their days with other individuals without collars because the study occurred during their mating season (Naganuma et al. 2021). Therefore, even if the scats were collected in clusters, it could not be determined that they were from the individuals being tracked. Therefore, in this study, we used the 102 scats collected as the representative diet of the bear population in this area.

We quantitatively analyzed scat using the point-frame method (Sato et al. 2000) to separate material from each sample into individual food categories. We placed scats on sieves (2 mm and 1 mm mesh) and washed the contents with tap water. We then spread the material remaining on the sieves onto an enamel tray with the bottom marked with a 5 mm × 5 mm grid and regarded the points of intersection as point frames. Four hundred points per scat were counted for each food category, as Sato et al. (2000) confirmed that this point-frame method could be used to accurately reflect volume.

We recorded the percent frequency and percent volume of each category in each sample. We calculated the percent frequency of occurrence (hereafter, fecal frequency) for each dietary item by dividing the frequency of occurrence by the total number of samples. We determined the percent frequency of occurrence and percent volume of each food item. The percent volume of each food item (hereafter, fecal volume) was estimated at one of five levels; <1%, 1% to 25%, 25% to 50%, 50% to 75%, or 75% to 100% and were assigned nominal values of 1%, 12.5%, 37.5%, 62.5%, and 87.5%, respectively. The calculations were as follows (Mealey 1980): Fecal frequency % = (number of scats containing that food item/total number of scats) × 100; Fecal volume % = total percent volume of that food item/total number of scats.

### Statistical analyses

To test our hypotheses, we conducted the following four analyses. All analyses were performed under R 4.0.3 (R Core Team 2018) and Supplementary Data SD1 shows a list of data used in each analysis.

First, for comparing results from video analysis and fecal analysis, we performed a Wilcoxon signed ranked test, using the coin package (Hothorn et al. 2008), to compare: (1) shooting relative frequency at 15-min intervals to fecal relative frequency; and (2) to fecal volume; and (3) average of the number of continuous clips to fecal relative frequency of each food items, because those data did not follow a normal distribution. We used a paired Student’s *t*-test to compare the average number of continuous clips to fecal volume, which both followed normal distributions. For comparison of shooting relative frequency to fecal relative frequency and fecal volume (“shooting relative frequency” vs. “fecal relative frequency” and “fecal volume”), we used six food items. For comparison of the average number of continuous clips to fecal relative frequency and fecal volume (“average of the number of continuous clips” vs. “fecal relative frequency” and “fecal volume”), we used five food items which could be identified correctly (Supplementary Data SD1). In other words, these food items are not included in the items that are considered unknown. In both comparison tests, we pooled the data of all four individuals.

Second, to evaluate the difference in average of the number of continuous clips of food items by video analysis, we use Kruskal–Wallis test using the “kruskal.test” function, followed by multiple pairwise comparisons of the food items using the Dunn’s test with the Benjamini–Hochberg correction of FSA package (Ogle et al. 2021). We used 16 food items and pooled data of all individuals (Supplementary Data SD1) in the analyses.

Third, in order to explore if clip shooting interval times (15, 30, 60, and 120-min intervals) change the frequencies of different food items, we used permutational multivariate analysis of variance (PERMANOVA) on a Bray–Curtis dissimilarity matrix with 9,999 permutations using the “adonis” function in the vegan package (Oksanen et al. 2020). We used shooting relative frequency of 24 food items as response variables at each time between clips and each individual (Supplementary Data SD1), with the time between clips as the explanatory variable. After the PERMANOVA, we used analyses of variance (ANOVAs) separately for each of the food items to evaluate the effect of the time between clips changes on the detection of each food item.

Finally, to understand differences in diet composition among individuals, we compared the shooting relative frequency of 23 food items documented with video analysis among individuals using a principal component analysis (PCA). Bear cubs were excluded from the analysis because they are unlikely to be a common food source and actual purpose may be infanticide. PCA ordinates the diet of each individual and represents variation within the composition of 23 food items, which can reduce the dimensionality of data sets and increase interpretability while minimizing information loss. Thus, the PCA provides us with differences among individuals by the location of data points and shows which food items explain individual diet by the loadings of each principal component.

## Results

### Food habits from video analysis

In total, we equipped four Asian black bears with camera collars (two 4-year-old males, a 12-year-old female, and a 4-year-old female) for an average of 41.3 days (Naganuma et al. 2021; Table 1). The total number of clips per bear was somewhat less than the theoretical maximum number of clips for the observation period—average success rate = 99.4%; range = 99.2% (Female A) to 99.7% (Female B) because the cameras occasionally failed to work properly, resulting in nothing being recorded on some clips. However, the cessation of camera operation was only temporary, and there was no catastrophic failure that would cause a complete cessation of data collection. We also did not document any errors in the camera components or programming/duty cycle. The rates of “foraging” clips varied among the individual bears, accounting for 6.6 ± 7.4% (Female A) to 23.9 ± 22.6% (Male B; the mean daily proportion of time ± *SD*), demonstrating a large interindividual variation in each of the behavior types (Table 1).

**Table 1.** —Age, weight, mean daily proportion of time spent on each activity (%; *SD*), total number of clips, and monitoring period of all individual Asian black bears in Okutama mountains, in 2018.

	Female A	Female B	Male A	Male B
Age	4	12	4	4
Weight	52 kg	42 kg	48 kg	42 kg
Traveling by land	5.5 ± 8.3	11.3 ± 9.5	20.0 ± 13.4	14.2 ± 8.7
Sleeping	42.0 ± 25.1	37.3 ± 16.8	22.4 ± 14.8	25.7 ± 17.9
Resting	7.9 ± 6.1	12.7 ± 7.0	9.4 ± 7.6	10.0 ± 8.0
Foraging	6.6 ± 7.4	12.3 ± 12.8	14.3 ± 11.5	23.9 ± 22.6
Sniffing something	8.5 ± 6.1	12.3 ± 9.1	17.2 ± 9.0	9.2 ± 6.7
Other	1.2 ± 1.9	2.4 ± 2.0	0.7 ± 1.3	1.6 ± 2.5
Unclear	28.3 ± 20.4	11.8 ± 10.1	15.9 ± 12.6	15.4 ± 13.4
Total number of clips	1,857 (36 days)	2,074 (40 days)	2,326 (45 days)	2,273 (44 days)
Monitoring period	11 June to 16 July	1 June to 10 July	25 May to 8 July	4 June to 17 July

**Fig. 2.**
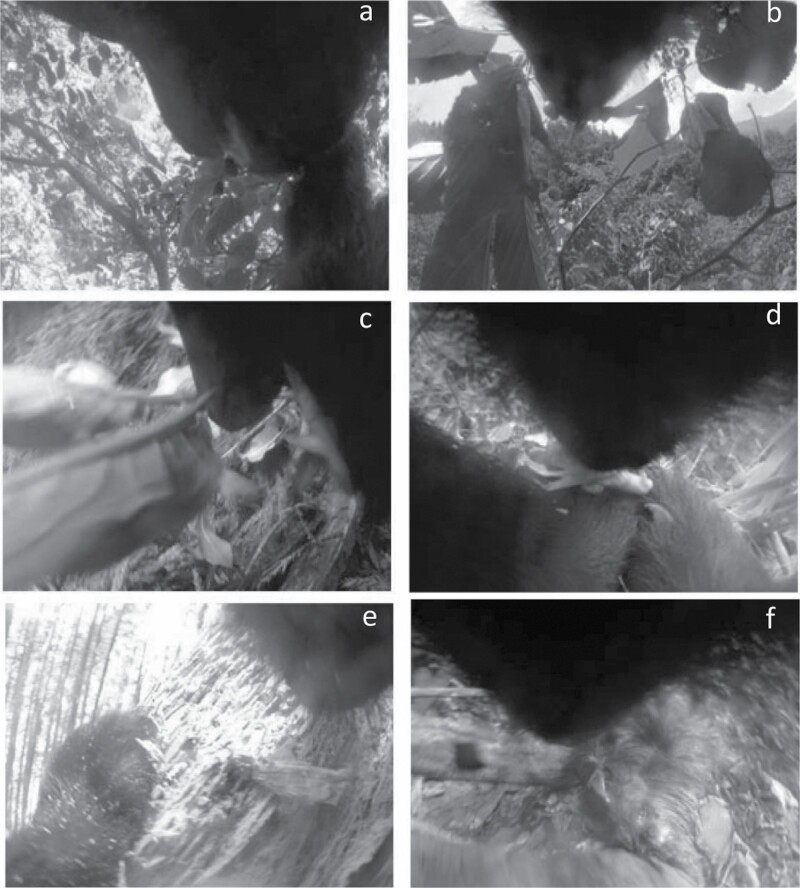
—Examples of video clips of bears eating some food items: (a) *Morus australis* fruits; (b) *Cerasus verecunda* fruits; (c) *Arisaema* spp.; (d) *Sasa* spp.; (e) the nest of Formicidae, destroying decayed tree; (f) *Capricornis crispus*.

Among the identified ingested food items from foraging behavior clips (Fig. 2, Supplementary Data SD2), most of the herbaceous plants, and leaves or flowers of woody plants, could be identified to the levels of species or genus (Table 2). As for soft mast and hard mast, we could identify some *Cerasus* species and *Morus bombycis*, and fallen *Quercus* acorns at the forest floor. Among mammals, we were able to confirm foraging on sika deer (*Cervus nippon*) including fawns and adult females, adult Japanese serows (*Capricornis crispus*), and an Asian black bear cub (thought to be infanticide by the adult male bear). We also confirmed bears eating *Vespula flaviceps* and *Bombus ardens*, both of which live in nests in the soil or in the trees, in the foraging behavior clips. In roughly 24% of clips showing foraging behavior, the food items could not be identified because the time duration of the acquired clips was too short. For example, the clips showed the scenes where the bear inserted and moved its mouth in a pile of fallen leaves or a decayed tree, or the clips showed the scenes where the bear had already eaten any food items in their mouth before the clip started (Table 2).

**Table 2.** —Percent relative frequency of occurrence (fecal frequency %: FF) and mean percent volume (fecal volume %: FV) for each food item found in the fecal samples and percent occurrence relative frequency during feeding video clips (clip frequency %: CF) of 1 min between clips and number of mean continuous clip (CC), mean number of continuous clips of foraging for same food items at the same location when the 15 min between clips shown in the video clip samples of Asiatic black bears in the Okutama mountains. We used pool data of all individuals.

	Fecal analysis		Video analysis (total)	
	FF	FV	CF	CC (range)
Plant material				
Green vegetation				
Grass plants				
Brassicaceae spp.			0.1	
*Sasa* spp.	5.0	92.0	0.4	1 (1)
*Arisaema* spp.	1.3	0.5	0.8	1 (1)
*Carpesium abrotanoides*			0.1	
*Boehmeria japonica*			1.2	1 (1)
Unknown	1.3	16.8	2.6	
Woody plants (leaves)				
*Cerasus sargentii*			2.0	1 (1)
*Cerasus verecunda*			1.8	1 (1)
*Cerasus* spp.	15.0	65.5		
*Pueraria montana*			0.5	1 (1)
*Castanea crenata*			0.1	
*Actinidia polygama*			0.8	1.4 ± 0.5 (1–2)
*Actinidia arguta*			1.0	1.4 ± 0.7 (1–3)
*Celastrus orbiculatus*			0.1	
*Magnolia obovata*			0.1	
Unknown			0.4	
Unknown	67.5	64.5		
Soft mast				
*Cerasus sargentii*	26.3	58.2	26.6	2.0 ± 1.3 (1–7)
*Cerasus verecunda*	25.0	59.0	21.8	1.5 ± 1.0 (1–6)
*Cerasus* spp.			2.0	1.2 ± 0.6 (1–4)
*Morus australis*	5.0	32.1	3.0	1.6 ± 1.0 (1–4)
*Prunus mume*	1.3	6.0		
*Prunus salicina*	1.3	69.9	2.7	1.3 ± 0.4 (1–2)
Hard mast				
*Juglans mandshurica*	1.3	1.8	0.2	1 (1)
*Quercus* spp.			0.6	1.7 ± 0.5 (1–2)
Unknown	5.0	8.4		
Others				
Flower: Ericaceae spp.			0.1	
Flower: *Actinidia polygama*			0.1	
Flower: *Actinidia arguta*			0.1	1 (1)
Flower: unknown	6.3	8.7		
Bark: conifer tree	1.3	1.4	4.4	1.2 ± 0.5 (1–3)
Animal material				
Insect				
*Vespula flaviceps*			0.1	
*Vespula* spp.	11.3	34.2		
*Bombus ardens*			0.1	
*Bombus* spp.	1.3	52.0		
Formicidae spp.	55.0	6.0	1.0	1 (1)
Hymenoptera spp.	2.5	5.7		
Coleoptera spp.	3.8	2.3		
Others				
*Capricornis crispus*: adult			2.3	2.2 ± 1.0 (1–4)
*Cervus nippon*: adult female			2.1	1.8 ± 1.2 (1–5)
*Cervus nippon*: fawn			1.3	1.8 ± 0.7 (1–3)
*Cervus nippon*	16.3	30.9		
*Ursus thibetanus*: cub			0.1	
*Ursus thibetanus*	6.3	24.7		
Unknown				
Unknown items				
Something in litter			11.3	
Something in decaying log			1.7	
Unknown	6.3	38.7	6.5	
No. of feces and foraging clips	102		843	

The duration of the average of the number of continuous clips for each food item was about one in most of the foods (Table 2), but there were some exceptions and using a Kruskal–Wallis test we found that there were significant differences in the number of continuous clips among food items (*P* < 0.001). Using multiple pairwise comparisons with the Dunn’s test, we found there were significant differences in number of continuous clips for the following pairs: *Boehmeria japonica* leaves vs. *C. crispus* (*Z* = −3.036, *P* = 0.048), *C. crispus* vs. *C. sargentii* leaves (*Z* = 3.345, *P* = 0.049), *C. sargentii* leaves vs. *C. sargentii* mast (*Z* = −3.283, *P* = 0.030), *C. crispus* vs. *C. verecunda* mast (*Z* = 2.954, *P* = 0.047), *C. sargentii* mast vs. *C. verecunda* mast (*Z* = 4.247, *P* = 0.003), *C. crispus* vs. conifer tree bark (*Z* = 3.021, *P* = 0.043), and *C. sargentii* mast vs. conifer tree bark (*Z* = 3.334, *P* = 0.034; Supplementary Data SD3).

**Fig. 3.**
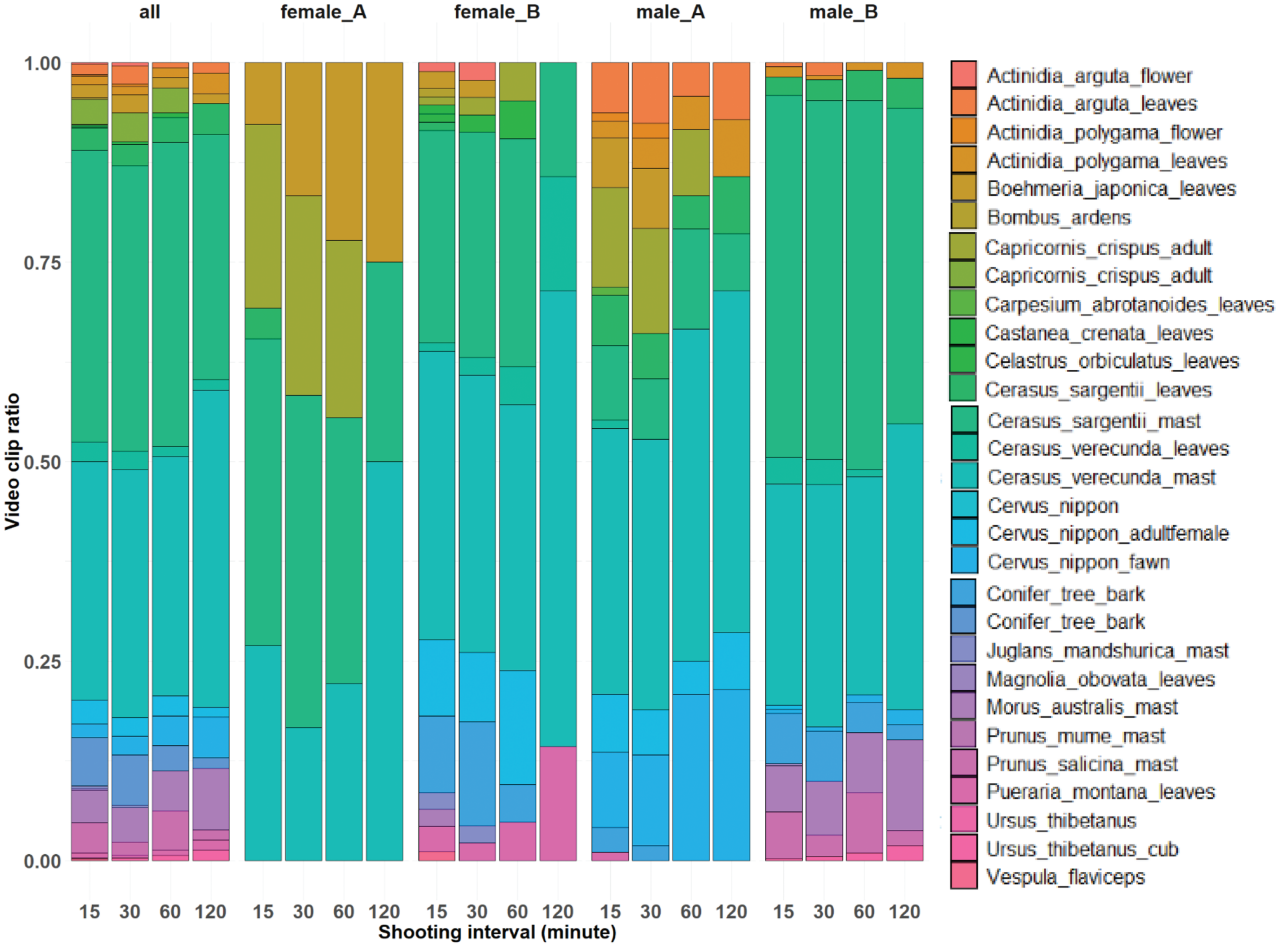
—Stacked bar plots with proportions of each video clip of each food item based on total number of video clips for pooled and all individuals (Female A, Female B, Male A, and Male B).

Using a MANOVA, we found the shooting relative frequency of food items was significantly different when accounting for variation in the time between clips (Table 3; Fig. 3; Supplementary Data SD4). We found a significant difference in shooting relative frequency of adult *C. crispus* by the time between clips increase (ANOVA; *F*_1,14_ = 4.638, *P* = 0.049; Supplementary Data SD5). In addition, shooting relative frequency of *B. japonica* leaves, conifer tree bark, and *C. nippon* adult female showed weakly significant difference in the time between clips increase (*B. japonica* leaves, *F*_1,14_ = 4.342, *P* = 0.056; conifer tree bark, *F*_1,14_ = 3.867, *P* = 0.069; *C. nippon* adult female, *F*_1,14_ = 4.519, *P* = 0.052; Supplementary Data SD5).

**Table 3.** —Permutational multivariate analysis of variance (PERMANOVA) results for video clip frequencies of food items by shooting interval increase based on Bray–Curtis distances.

Source of variation	d.f.	SS	MS	*F*	*R*²	*P*-value
Shooting interval	1	0.703	0.703	3.665	0.207	0.012
Residuals	14	2.684	0.192		0.793	
Total	15	3.387			1	

SS, sum of squares; MS, mean sum of squares.

### Food habits from fecal analysis

Among the plant materials, most of the soft mast items were identified to species (fecal frequency of “Unknown” = 2.5%), while species of the all leaves of herbaceous plants and woody plants were not possible for us to identify (fecal frequency of “Unknown” = 67.5%; Table 2). Among the animal materials, we could confirm mammals as food items only from feces that included hair to identify deer and bear species. Species of most insects were also difficult to identify; however, we detected small amounts of ants (Formicidae) in 55% (fecal frequency) of the scat samples. In the fecal frequency, 6.3% of the digested foods could not be identified in the scat contents.

### Comparison of food habit methods

For comparison between results from our video analyses and scat analyses, we found that there were significant differences in shooting relative frequency vs. fecal relative frequency (*Z* = 2.279, *P* = 0.023) and shooting relative frequency vs. fecal volume (*Z* = 2.589, *P* = 0.010). Averages of the number of continuous clips and fecal relative frequency were not significantly different (*Z* = 1.683, *P* = 0.092), but averages of the number of continuous clips and fecal volume were significantly different (*t* = 3.463, d.f. = 4, *P* = 0.026). In terms of specific food items, we could not identify any herbaceous or woody leaves through fecal analysis and could only identify three genera, but video analysis showed 10 species and three genera. Similarly, using video analysis we identified three species of flowers, but could not identify any species with fecal analysis. Among soft mast, we identified five species from the seeds with fecal analysis and identified four species with video analysis. These four species all had a high percent relative frequency of occurrence or high mean percent volume by fecal analysis. Among the insects, we identified a large amount of Formicidae spp. in fecal analysis; however, we were seldom able to identify it in video analysis. Among the mammals, when using fecal analysis, we could only identify to the mammal species, but the video analysis allowed us to identify additional attributes (e.g., growth stage and sex) of the species.

### Individual differences of food habits assessed by video analysis

**Fig. 4.**
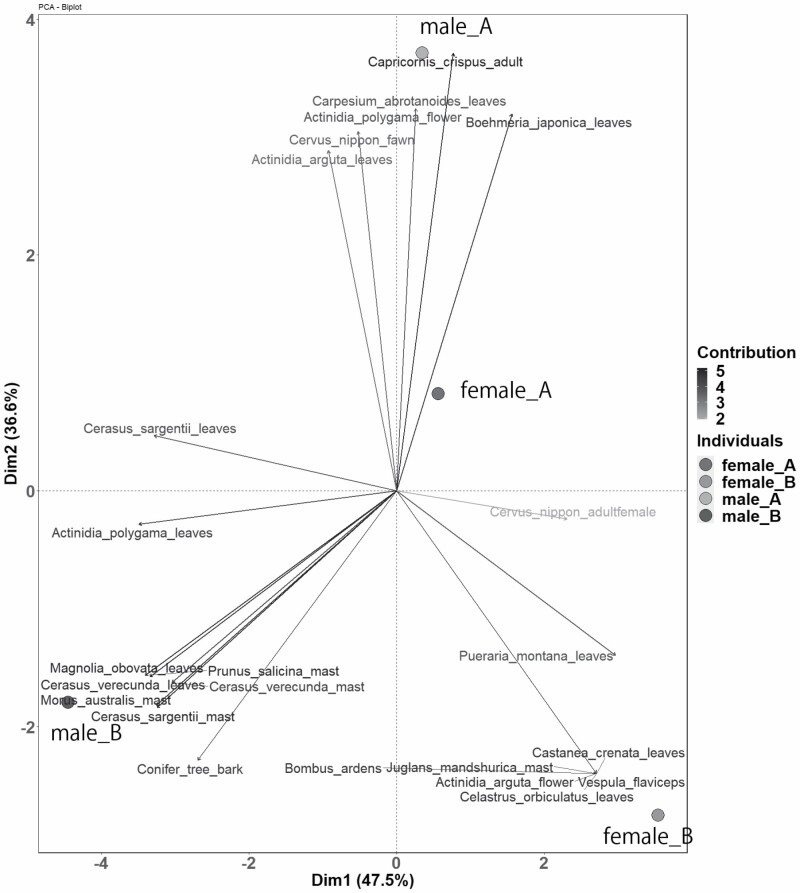
—Principal component analysis (PCA) of diet composition for four individuals from video analyses. The PCA biplot showing the scores of each individual as points, and the contribution of variables to the principal components as arrows (with black representing high contributions, gray representing low contributions). The length of the arrows approximates the variance of the variables, whereas the angles between them approximate their correlations, and distances between points represent the similarities between individuals.

The video analyses revealed differences in the foraging items among individuals. When performing PCA for shooting relative frequency of 23 food items of four individuals, the first two PCs could explain 84.1% of total variation in shooting relative frequency of all food items (Supplementary Data SD4 and SD6; Fig. 4). Variables for leaves of *C. sargentii*, *C. verecunda*, *A. polygama*, and *M. bovate*; soft masts of *C. sargentii*, *M. australis*, and *P. salicina*; and *U. thibetanus* cub have highly negative loadings (loading < −0.25) on PC1 (Supplementary Data SD6; Fig. 4). PC1 partitioned individuals into Male B and the others, with the diet of Male B containing more of the above food items with high contributing values (Fig. 4). Variables for leaves of *C. abrotanoides*, *B. japonica*, *A. arguta*, and *A. polygama* flower; *C. crispus* adult; and *C. nippon* fawn had high positive loadings (loading > 0.25) on PC2 (Supplementary Data SD6; Fig. 4). PC2 partitioned individuals into Male A/Female A and Male B/Female B, and diets of Male A/Female A include more of the above food items with high contributing values (Fig. 4). Male A and Female A are located relatively closer, but the points of all four individuals were separated from one another (Fig. 4).

## Discussion

In this study of Asian black bear foraging, we contrasted the novel method of using animal-borne video systems with the traditional method of analyzing bear feces. From these comparisons, we found that each method offered both advantageous and disadvantageous aspects for estimating food habits of large carnivores. Our first objective was to compare both methods and to examine the characteristics of food items we could confirm in the diet of bears using each method. As we expected, the video analysis was able to identify most of the types of herbaceous and woody leaves. In addition, we were able to identify the attributes (sex and growth stage) of the mammals that were eaten and even the species of insects. In contrast, in the fecal analyses the easily digestible herbaceous and woody plants and animal materials were difficult to identify, supporting our first hypothesis.

When the results of both analyses were compared, however, a higher shooting frequency did not necessarily mean a higher fecal relative frequency or fecal volume. Possible reasons for these results include the following. The video analysis shows only the foods ingested by bears in the short periods when the camera collar is recording (10-s clips at 15-min intervals). For this reason, the camera is less likely to record food items that are infrequently ingested or food items that are quickly ingested. For fecal analysis, the gut retention time of bears is 4–36 h and there are multiple defecations per day (Koike et al. 2011). Accordingly, one scat sample is likely to contain several different food items ingested over a period of several hours. Thus, fecal analysis would also be more likely to detect food items that are eaten incidentally or intentionally only infrequently.

Food items for which bears spent a longer time foraging (longer number of continuous clips) had a higher fecal relative frequency in the fecal analysis. Prior studies using fecal analysis have reported that the soft mast of *Cerasus* are one of principal foods in this season (Koike 2010; Furusaka et al. 2017). Presence of the soft mast of *Cerasus* was confirmed to be consumed by bears at a high relative frequency by both the methods and when using longer continuous clips. Thus, it may be that the differences in diversity and frequency of food items between both methods are associated with: (a) differences in frequency that the food items are ingested (principal food or unfrequently ingested foods); (b) the time needed to consume the food; and (c) how the food is influenced by chewing and digestion.

There were also issues with the video analysis, including that the use of fecal analysis was much better at identifying bears consuming ants and beetles (Coleopteran) than the collar cameras. The heads of ants and the exoskeletons of *Scarabaeidae* and *Carabinae* were confirmed by fecal analysis, but almost no scenes when bears ingested these kinds of food items could be confirmed in the clips. There were many clips where bears inserted and moved their mouth in a pile of fallen leaves or decayed trees, which may have been bears ingesting these food items, because it is known that bears consume these insects (Koike et al. 2012). These clips indicate the difficulty in identifying small food items such as ants and beetles on the clips, even though fecal analysis shows that they are frequently consumed.

For our second objective, we examined the characteristics of food items that would be less likely to be captured by increasing the time between clips in future studies. The results showed that the shorter the time between clips, the greater number of food items that were documented. In addition, the food items with certain rates of shooting relative frequency and a longer average of the number of continuous clips at the 15-min interval tended to appear even when the time between clips was extended. On the other hand, there were some food items that decreased with shooting relative frequency, including serows, adult deer, conifer bark, and leaves of *B. japonica* (which are likely to be accidental foraging; Koike 2010; Inagaki et al. 2020). In our initial hypothesis 2, we assumed that food items with a low shooting relative frequency and short average of the number of continuous clips at 15-min intervals would be less likely to be detected when the time between clips was increased, but although these were basically sufficient to explain the results, we were unable to explain the comparative frequency of some of the food items. Therefore, hypothesis 2 was only partially supported.

Finally, as an application of the video analysis, we examined the possibility of discriminating the presence or absence of individual differences in feeding habits. The differences in food items documented in the clips and the results of PCA analysis showed that the diet of Male B was different from that of the other three individuals. Among the food items that caused the difference was soft mast of *C. sargentii*, one of the stable food items of the season (Koike et al. 2008a; Koike 2009, 2010). In addition, the diet of Male A included tree leaves, serows, and deer. Although the sample size was very small, our study supports the finding that the feeding habits of bears potentially vary among individuals (Naganuma et al. 2020), and shows that video analysis can be an important method for revealing individual differences in diet.

Analysis of food habits using camera collars seems to be useful in recognizing the principal foods and confirming ingested foods, which are often difficult to identify to species by fecal analysis. On the other hand, it also does not seem possible to correctly identify small food items found and ingested during movement to another place or minute foods such as insects. Additionally, it is difficult to acquire clips at night using the existing camera collar system, and the current memory storage and battery life limit the amount of data that studies can collect (i.e., diet can be estimated for each individual over a 2- to 4-week period depending on the video acquisition schedule). As a result, these become “snapshots” of feeding habits that may or may not be reflective of seasonal and annual feeding habits as influenced by plant phenology. Furthermore, sometimes mud or fallen leaves would stick to the camera lens, making it impossible to capture images, or the camera would rotate its head, making it impossible to capture the tip of the mouth. Accordingly, with the current level of camera collar technology, it is not a suitable method for correctly identifying all the food items in the diet. Camera collars appear to be useful, however, as an auxiliary method to supplement other existing techniques such as fecal analysis, to improve the accuracy of analysis of bear food habits and foraging behavior.

This method can also help with emerging human–bear conflicts. As the distribution of Asian black bears in most areas of Japan expands, large numbers of bears expand into urban areas every few years, and many of them are caught and killed. However, the cause of this behavior, especially in summer, is unknown and is a major problem for bear management in Japan. In the future, surveys using camera collars on bears living around urban areas may lead to the elucidation factors motivating their penetration into urban areas.

There are some important aspects to consider when beginning future studies using collar cameras. First, the video collars used in this study did not have fatal malfunctions (average success rates were 99.4%), thus being a promising tool for data collection. Second, another advantage is that fecal analysis requires a certain amount of experience to estimate fecal contents from intact fecal residues, which can lead to less accuracy. On the other hand, video analysis also requires some familiarity, but since it is possible to identify food items that have retained their original form it is possible for more investigators to identify the same items accurately. In addition, it takes 30 min to an hour, depending on the experience and accuracy of the person, to wash a single scat and identify its contents, but with video analysis, a single sample can be processed in one playback. Fourth, comprehensive analysis of the clips acquired by camera collars could also be combined with GPS data or other sensors. For example, it is generally believed that bears tend to ingest food items at clusters of GPS locations (Rauset et al. 2012), but these field surveys overlook the foods ingested quickly or while they are moving, as well as food items not aggregated enough to become a cluster. Combining clips of the foraging behavior of bears at the sites between clusters may lead to new and more accurate findings. Thus, the ability to link behavioral information with detailed dietary information on an individual basis is an advantage of using a camera collar, and is probably a special feature not found in DNA analysis of fecal contents or stable isotope analysis of body hair.

## Supplementary Data

Supplementary data are available at *Journal of Mammalogy* online.

**Supplementary Data SD1.**—List of food items used in all statistical analyses.

**Supplementary Data SD2.**—Examples taken from video clips recorded during foraging.

**Supplementary Data SD3.**—The results of Dunn’s test comparing number of continuous video clips of food items.

**Supplementary Data SD4.**—Percent occurrence relative frequency during feeding video clips (shooting frequency) of several times between clips (15, 30, 60, and 120-min intervals) in pooled of all individuals. And, percent of occurrence relative frequency of each food item during feeding video clips (shooting frequency in 15 min between clips) in Female A, Female B, Male A, and Male B of Asiatic black bears in the Okutama mountains.

**Supplementary Data SD5.**—Analysis of variance (ANOVA) results for percent of occurrence frequency during feeding video clips of each food item when increasing the time between clips.

**Supplementary Data SD6.**—Results of the principal component analysis (PCA) based on percent of occurrence frequency during feeding video clips of food items for four individuals.

gyac101_suppl_Supplementary_Data_SD1

gyac101_suppl_Supplementary_Data_SD2

gyac101_suppl_Supplementary_Data_SD3

gyac101_suppl_Supplementary_Data_SD4

gyac101_suppl_Supplementary_Data_SD5

gyac101_suppl_Supplementary_Data_SD6

## References

[CIT0001] Arthur K.E. , O’Neil J.M., Limpus C.J., Abernathy K., Marshall G. 2007. Using animal-borne imaging to assess green turtle (*Chelonia mydas*) foraging ecology in Moreton Bay, Australia. Marine Technology Society 41:9–13.

[CIT0002] Beringer J. , Millspaugh J.J., Sartwell J., Woeck R. 2004. Real-time video recording of food selection by captive white-tailed deer. Wildlife Society Bulletin 32:648–654.

[CIT0003] Bowen W.D. , Tully D., Boness D.J., Bulheier B.M., Marshall G.J. 2002. Prey-dependent foraging tactics and prey profitability in a marine mammal. Marine Ecology Progress Series 244:235–245.

[CIT0004] Bowersock N. , Gunther K.A., Wyman T., Dickinson C., Bergum D., van Manen F., Haroldson M. 2015. Camera collars: the evolution of tracking bears through Yellowstone. Yellowstone Science 23:68–71.

[CIT0005] Brockman C.J. , Collins W.B., Welker J.M., Spalinger D.E., Dale B.W. 2017. Determining kill rates of ungulate calves by brown bears using neck-mounted cameras. Wildlife Society Bulletin 41:88–97.

[CIT0006] Burkholder D.A. , Heithaus M.R., Thomson J.A., Forqurean J.W. 2011. Diversity in trophic interactions of green sea turtles *Chelonia mydas* on a relatively pristine coastal foraging ground. Marine Ecology Progress Series 439:277–293.

[CIT0007] Furusaka S. , Kozakai C., Nemoto Y., Umemura Y., Naganuma T., Yamazaki K., Koike S. 2017. The selection by the Asiatic black bear of spring plant food items according to their nutritional values. Zookeys 672:121–133. 10.3897/zookeys.672.10078 PMC5527342 28769668

[CIT0008] Heithaus M. , McLash J.J., Frid A., Dill L.M., Marshall G.J. 2002. Novel insights into green sea turtle behaviour using animal-borne video cameras. Journal of the Marine Biological Association of the United Kingdom 82:1049–1050.

[CIT0009] Hernandez S.M. , Loyd K.A.T., Newton A.N., Carswell B.L., Abernathy K.J. 2018. The use of point-of-view cameras (Kittycams) to quantify predation by colony cats (*Felis catus*) on wildlife. Wildlife Research 45:357–365.

[CIT0010] Hothorn T. , Hornik K., van de Wiel M.A., Zeileis A. 2008. Implementing a class of permutation tests: the coin package. Journal of Statistical Software 28:1–23.27774042

[CIT0011] Inagaki A. , Allen M., Maruyama T., Yamazaki K., Tochigi K., Naganuma T., Koike S. 2020. Vertebrate scavenger guild composition and utilization of carrion in an East Asian temperate forest. Ecology and Evolution 10:1223–1232. 32076509 10.1002/ece3.5976 PMC7029075

[CIT0012] Japan Meteorological Agency. 2021. Past weather data research. Tokyo, Japan (in Japanese). http://www.data.jma.go.jp/gmd/risk/obsdl/index.php. Accessed 29 April 2021.

[CIT0013] Koike S. 2009. Fruiting phenology and its effect on fruit feeding behavior of Asiatic black bears. Mammal Study 34:47–52.

[CIT0014] Koike S. 2010. Long-term trends in food habits of Asiatic black bears in the Misaka Mountains on the Pacific coast of central Japan. Mammalian Biology 75:17–28.

[CIT0015] Koike S. , Kasai S., Yamazaki K., Furubayashi K. 2008a. Fruit phenology of *Prunus jamasakura* and the feeding habit of the Asiatic black bear as a seed disperser. Ecological Research 23:385–392.

[CIT0016] Koike S. , Masaki T., Nemoto Y., Kozakai C., Yamazaki K., Kasai S., Nakajima A., Kaji K. 2011. Estimate of the seed shadow created by the Asiatic black bear (*Ursus thibetanus*) and its characteristics as a seed disperser in Japanese cool-temperate forest. Oikos 120:28080–28290.

[CIT0017] Koike S. , Morimoto H., Goto Y., Kozakai C., Yamazaki K. 2008b. Frugivory of carnivores and seed dispersal of fleshy fruits in cool-temperate deciduous forests. Journal of Forest Research 13:215–222.

[CIT0018] Koike S. , Morimoto H., Goto Y., Kozakai C., Yamazaki K. 2012. Insectivory by sympatric five carnivores in cool-deciduous forests. Mammal Study 37:73–83.

[CIT0019] Koike S. , Nakashita R., Naganawa K., Koyama M., Tamura A. 2013. Changes in diet of a small, isolated bear population over time. Journal of Mammalogy 94:361–368.

[CIT0020] Kooyman G. 2007. Animal-borne instrumentation systems and the animals that bear them: then (1939) and now (2007). Marine Technology Society Journal 41:6–8.

[CIT0021] Lavelle M.J. , Blass C.R., Fischer J.W., Hygnstrom S.E., Hewitt D.G., VerCauteren K.C. 2015. Food habits of adult male white-tailed deer determined by camera collars. Wildlife Society Bulletin 39:651–657.

[CIT0022] Lavelle M.J. , Hygnstrom S.E., Hildreth A.M., Campbell T.A., Long D.B., Hewitt D.G., Beringer J., VerCauteren K.C. 2012. Utility of improvised video-camera collars for collecting contact data from white tailed deer: possibilities in disease transmission studies. Wildlife Society Bulletin 36:828–834.

[CIT0023] Litvaitis J.A. 2000. Investigating food habits of terrestrial vertebrates. In: Boitani L., Fuller T.K., editors. Research techniques in animal ecology: controversies and consequences. Columbia University Press, New York, USA; p. 165–190.

[CIT0024] Loyd K.A.T. , Hernandez S.M., Carroll J.P., Abernathy K.J., Marshall G.J. 2013. Quantifying free-roaming domestic cat predation using animal-borne video cameras. Biological Conservation 160:183–189.

[CIT0025] Mammal Society of Japan. 2009. The guidelines for use and collection of animals in research. https://www.mammalogy.jp/guideline.html. Accessed 25 May 2021 (in Japanese).

[CIT0026] Mealey S.P. 1980. The natural food habits of free-ranging grizzly bears in Yellowstone National Park, 1973–1974. Bears: Their Biology and Management 4:281–292.

[CIT0027] Moll R.J. , Millspaugh J.J., Beringer J., Sartwell J., He Z. 2007. A new ‘view’ of ecology and conservation through animal-borne video systems. Trends in Ecology and Evolution 22:660–668. 18006184 10.1016/j.tree.2007.09.007

[CIT0028] Morrison M.L. , Marcot B.G., Mannan R.W. 1998. Wildlife–habitat relationships—concepts and applications. Island Press, Washington, District of Columbia, USA.

[CIT0029] Naganuma T. , Koike S., Nakashita R., Kozakai C., Yamazaki K., Furusaka S., Kaji K. 2020. Age- and sex-associated differences in the diet of the Asian black bear: importance of hard mast and sika deer. Mammal Study 45:155–166.

[CIT0030] Naganuma T. , Nakashita R., Tochigi K., Zedrosser A., Kozakai C., Yamazaki K., Koike S. 2022. Functional dietary response of Asian black bears to changes in sika deer density. The Journal of Wildlife Management 86:e22218.

[CIT0031] Naganuma T. , Tanaka M., Teduka S., Steyaert S.M.J.G., Tochigi K., Inagaki A., Myojo H., Yamazaki K., Koike S. 2021. Animal-borne video systems provide insight into the reproductive behavior of the Asian black bear. Ecology and Evolution 11:9181–9190. 10.1002/ece3.7722 PMC8293739 34306614

[CIT0032] Newmaster S.G. , Thompson I.D., Steeves R.A.D., Rodgers A.R., Fazekas A.J., Maloles J.R., McMullin R.T., Fryxell J.M. 2013. Examination of two new technologies to assess the diet of woodland caribou: video recorders attached to collars and DNA barcoding. Canadian Journal of Forest Research 43(10):897–900. doi:10.1139/cjfr-2013-0108

[CIT0033] Ogle D.H. , Wheeler P., Dinno A. 2021. FSA: fisheries stock analysis. R package version 0.8.32. https://github.com/droglenc/FSA. Accessed 15 April 2021.

[CIT0034] Oksanen J. , Blanchet F.G., Kindt R., Legendre P., Minchin P.R., O’Hara R.B., Simpson G.L., Solymos P., Stevens M.H.H., Wagner H. 2020. vegan: community ecology package. R package version 2.5-7. https://CRAN.R-project.org/package=vegan. Accessed 25 April 2021.

[CIT0035] Okutama Town. 2014. Condition of environment. In: Okutama town environment basic plan. Okutama Town Office, Okutama, Japan; p. 7–48 (in Japanese).

[CIT0036] Pagano A.M. , Durner G.M., Rode K.D., Atwood T.C., Atkinson S.N., Peacock E., Costa D.P., Owen M.A., Williams T.M. 2018. High-energy, high-fat lifestyle challenges an Arctic apex predator, the polar bear. Science 359:568–572. 29420288 10.1126/science.aan8677

[CIT0037] R Core Team. 2018. R: a language and environment for statistical computing. R Foundation for Statistical Computing, Vienna, Austria.

[CIT0038] Rauset G.R. , Kindberg J., Swenson J.E. 2012. Modelling female brown bear kill rates on moose calves using global positioning satellite data. The Journal of Wildlife Management 76:1597–1606.

[CIT0039] Sato Y. , Mano T., Takatsuki S. 2000. Applicability of the point-frame method for quantitative evaluation of bear diet. Wildlife Society Bulletin 28:311–316.

[CIT0040] Takahata C. , Takii A., Izumiyama S. 2017. Season-specific habitat restriction in Asiatic black bears, Japan. The Journal of Wildlife Management 81:1254–1265.

[CIT0041] Viejou R. , Avgar T., Brown G.S., Patterson B.R., Reid D.E.B., Rodgers A.R., Shuter J., Thompson I.D., Fryxell J.M. 2018. Woodland caribou habitat selection patterns in relation to predation risk and forage abundance depend on reproductive state. Ecology and Evolution 8:5863–5872. 29938099 10.1002/ece3.4124 PMC6010817

[CIT0042] Watanabe Y.Y. , Takahashi A. 2013. Linking animal-borne video to accelerometers reveals prey capture variability. Proceedings of the National Academy of Sciences of the United States of America 110:2199–2204. 23341596 10.1073/pnas.1216244110 PMC3568313

[CIT0043] Wilmers C.C. , Nickel B., Bryce C.M., Smith J.A., Wheat R.E., Yovovich V. 2015. The golden age of bio-logging: how animal-borne sensors are advancing the frontiers of ecology. Ecology 96:1741–1753. 26378296 10.1890/14-1401.1

[CIT0044] Yamazaki K. , Kozakai C., Kasai S., Goto Y., Koike S., Furubayashi K. 2008. A preliminary evaluation of activity-sensing GPS collars for estimating daily activity patterns of Japanese black bears. Ursus 19:154–161.

